# “Medical student syndrome”: a real disease or just a myth?—a cross-sectional study at Menoufia University, Egypt

**DOI:** 10.1186/s43045-023-00312-6

**Published:** 2023-05-19

**Authors:** Huda A. Sherif, Khaled Tawfeeq, Zahraa Mohamed, Lobna Abdelhakeem, Sara H. Tahoon, Mahasen Mosa, Karima Samy, Karema Hamdy, Lamiaa Ellakwa, Salma Elnoamany

**Affiliations:** Faculty of Medicine, Menoufia University, Menoufia, Egypt

**Keywords:** Hypochondria, Nosophobia, Medical students

## Abstract

**Background:**

A widely held belief is that “Medical student syndrome” is frequently experienced by young medical students, that is, they experience the symptoms of the diseases they are studying or fear of having such illness. A hypothesis is that because medical students constantly learn about life-threatening conditions and diseases, they experience persistent fear and stress regarding having a severe medical condition, an anxiety-related illness called nosophobia.

**Results:**

Although medical students scored an average of 14.14 on a scale measuring potential nosophobia a, the difference between their scores and those of non-medical students, who scored an average of 0.11, is significantly higher (*p* 0.001). According to the presented analysis, non-medical and medical students exhibit distinct levels of nosophobia. The analysis of responses to hypochondriacal behaviors revealed that students from non-medical faculties scored an average of 1.43 points. By contrast, the average score for medical students was 7.87, which is significantly higher than that of the non-medical students (*p* 0.001).

**Conclusions:**

Medical students are at higher risk for health anxiety and hypochondrial attitudes than non-medical students are.

## Background

A widely held belief is that “Medical student syndrome” is frequently experienced by young medical students, that is, they experience the symptoms of the diseases they are studying or fear of having such illness [1].

A hypothesis is that because medical students constantly learn about life-threatening conditions and diseases, they are in persistent fear and stress of having a serious medical condition, an anxiety-related illness called nosophobia [1, 2]. Studying all these medical diseases and knowledge results in medical students applying them to themselves, making them very worried that they will become seriously ill such that they exaggerate minor symptoms, a condition called hypochondria [3]. The reasons behind the evolution of this phenomenon are the exposure to the symptoms of patients, who medical students are in frequent contact with, and the competitive, troublesome environment of medical schools [3–5].

To scientifically identify the psychosomatic terms used in our research, we used the following definitions: “nosophobia” is an uncontrollable fear of having a certain disease, and “hypochondria” is a persistent fear of having a serious condition emerging from delusions of contracting the disease and exaggerating minor symptoms despite the appropriate medical check-up and evaluation (Anon n.d. [2];). In addition, because medical students are exposed to severe stress, they are more likely to develop anxiety disorders and depression than non-medical students [3, 4]. Is medical student disease an actual condition, and does it occurs in the population?

The first description of medical student disease appeared in the 1960s. However, its scientific background is weak [1, 6]. A study conducted in the UK found no significant variance in the degrees of hypochondria and nosophobia between those who studied medicine and other students, which did not substantiate this developing occurrence [6]. Another observational study conducted in Poland concluded that medical students are more anxious about their health than non-medical students. Notably, students not studying medicine in Katowice had a higher proclivity for hypochondria and nosophobia symptoms than medical students [1]. However, due to the antithetical articles and the insufficient data on the situation in Egypt, we studied the student population at Menofia University to assess nosophobia and hypochondria in medical and non-medical students.

In addition, we considered the COVID-19 pandemic, which increased the stress in the population and the fear of contracting a disease. This finding also leads to social isolation and limited activities and distancing policies used to control disease dissemination, which impacts mental status, increasing stress, depression, and anxiety disorders [7, 8]. We also considered whether the pandemic affected the degree of nosophobia and hypochondriacal symptoms, how medical students managed these symptoms, and how to help those [1]**.**

## Methods

This study was an analytic cross-sectional study conducted among medical (*n* = 285) and non-medical (*n* = 97) students at Menoufia University. An online questionnaire was adapted from another study [1] and distributed through social medial platforms (Facebook groups, WhatsApp groups, Telegram, and LinkedIn) from February to March 2022.

We used the Raosoft sample size calculator for our sample. The result was (382) participants to fulfill the target of 95% CI, 5% margin of error, and a 5 response distribution.

### Study procedure and materials

The adapted questionnaire was translated into Arabic, the mother tongue in Egypt. Egyptian psychiatry experts assessed the language to assess the clarity and quality of translation. After that a pilot study was conducted to insure the validity of the questionnaire after translation. A Google Form was created, and participants were invited to complete and submit the form after the study’s nature and importance were explained.

The questionnaire had four sections: sociodemographic history, fear of disease, clinical history and thinking about the disease and a fear of COVID-19 that would lead to visiting doctors and attitudes about COVID-19. For all questions, we used a 5-item Likert scale (1 = *disagree totally* and 5 *agree totally*).

The sociodemographic data included age, gender, year of study, current financial situation, student of Menoufia University, medical student or not, and type of health care usually used.

To assess fear of disease, we asked about the fear of getting sick the, fear of microorganisms in the surroundings before and after COVID-19, and fear of illness that causes awakeness at night.

We assessed the clinical history that appeared on the patient and thought about it by asking some questions. For example, the questions asked about paying more attention to disease symptoms related to the patients' topics, panic attacks (palpitation, chest pain, shortness of breath, nausea) associated with fearing for one's health, suspecting disturbing symptoms, and thinking of symptoms that indicate a medical condition that one suspects in himself.

To assess the fear of COVID-19 that leads to visiting doctors, we asked about visiting the doctor when suspecting a self-diagnosed disease and trusting the doctor when he denies the condition you suspect.

For attitude about COVID-19, we asked the following: before COVID-19, did you wash your hands excessively for fear of microorganisms? Before and during COVID, did you use therapy due to your fear of getting sick? And what mental disorders do you suffer from?

### Ethical considerations

To ensure confidentiality, we acquired informed permission from the Institutional Review Board at Menoufia University’s Faculty of Medicine.

All survey participants completed a written informed consent form on the first page of the survey, and their approval was requested to complete the questionnaire. If the participant replied “yes” to the first question on the form, they consented to participate, and the survey began. No responder was compelled to participate in the survey, and their participation was contingent on their consent, which they could revoke at any moment. The obtained data remained private, and only authorized team members could access it. Furthermore, data were encrypted and coded for use primarily in statistical analysis via computer software.

### Data analysis

The presented analysis was conducted using Statistical 13.3. Alpha 0.05 was the significance level for the overall statistical summary. To find answers to the research questions, we used Student’s *t* tests and Spearman’s rho rank correlation coefficient to examine differences. In the additional analysis, the disparity among the groups of respondents required using a non-parametric Mann–Whitney *U* test. The comprehensive evaluation was supplemented by an analysis of response frequency and the results of the chi-square test of independence.

## Results

The sample was as follows: 382 students (females 201 (70.5%) for medical students and 54 (55.7%) for non-medical students. The medical student group comprised 285 students from the Faculty of Medicine’s various years. The non-medical student group comprised 97 students from all years of academic school who were not studying medicine (e.g., construction, administration, IT, pedagogy, law, management, philology). The mean age was 20.931 ± 1.668 for the medical group and 20.381 ± 1.531 for the non-medical group (Table 1).

**Table 1:** Cross-section of the group

	Medical students		Non-medical students		*χ*^2^	*P* value
	No.	%	No.	%		
Gender					7.197	0.007
Male	84	29.5%	43	44.3%		
Female	201	70.5%	54	55.7%		
Age (mean ± SD)	20.931 ± 1.668		20.381 ± 1.531			
Study group					75.995	0
First	31	10.9%	24	24.7%		
Second	67	23.5%	32	32.99%		
Third	64	22.5%	15	15.5%		
Fourth	18	6.3%	26	26.8%		
Fifth	99	34.7%	0	0.0%		
Sixth	6	2.1%	0	0.0%		
Financial condition					3.724	0.293
Bad	3	1.1%	1	1%		
Medium	71	24.9%	16	16.5%		
Good	120	42.1%	50	51.5%		
Very good	91	31.9%	30	30.9%		
Type of health service used					5.968	0.05
Government sector and health insurance	43	15.1%	25	25.77%		
Private health sector private clinics and hospitals	76	26.7%	20	20.6%		
Both services	166	58.2%	52	53.6%

**Table 2:** A statistical summary of the study group’s nosophobia variables: hypochondria, and pandemic anxiety

Variable under test	Min	Max	*M*	SD	SKE	*K*	*d*
Hypochondria symptoms	3	25	7.87	4.4	0.142	0.31	0.13*
Nosophobia symptoms	6	26	14.1	4.62	0.144	0.288	0.121*
Fear of pandemic							
Before COVID-19 pandemic	1	6	2.02	0.64	0.64	− 0.16	0.58*
During COVID-19 pandemic	0.81	5	1.01	0.31	0.31	− 0.46	0.11*

According to the table, we can say that the tested variables are consistent with the normal distribution and that the results of hypochondria and nosophobia symptoms are away from the mean, hypochondria symptoms SD = 4.4 and mean value of 7.87 and nosophobia symptoms SD = 4.62 and mean value of 14.1(Table 2).

Analyses were conducted for the number of observations. *Max*—maximum, *Min*—minimum, *K* Kurtosis, *M* Average, *SKE* Skewness, *SD* Standard deviation, *d* The value of the Kolmogorov–Smirnov test

*p < 0.05

Table 1 shows a significant difference between medical and non-medical students according to sex and age; however, no significant difference according to other demographic data was observed.

**Fig. 1:**
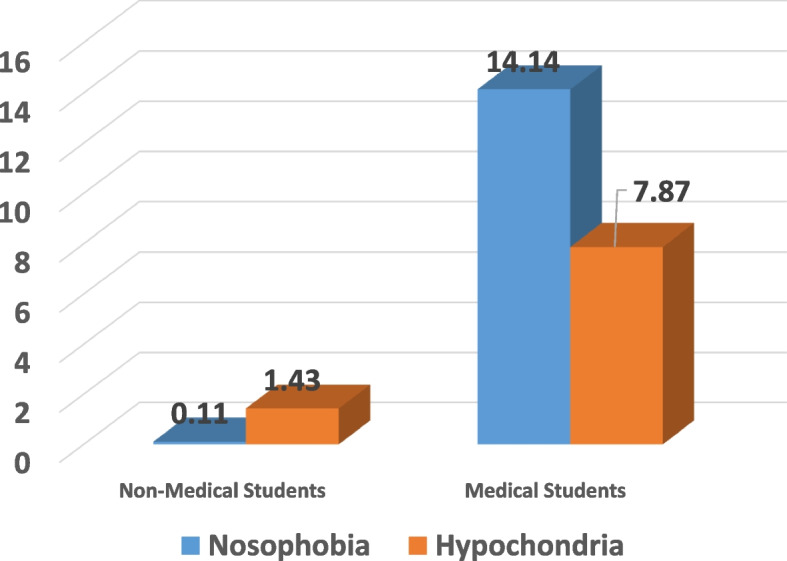
Student’s *t* test results for independent data regarding the level of nosophobia and hypochondria symptoms among non-medical (left) and medical (right) students

Table 3 illustrates the difference between non-medical and medical students' levels of nosophobia and hypochondria symptoms. Although the average grade for medical students was 14.14 on a scale measuring potential nosophobia, the difference between them and non-medical students, who scored an average of 0.11, was significantly higher (*p* 0.001). According to the presented analysis, non-medical and medical students experience varying degrees of nosophobia. The analysis of responses to hypochondriacal behaviors revealed that students from non-medical faculties scored an average of 1.43 points. By contrast, the average score for medical students was 7.87, which is significantly higher than that of the non-medical students (*p* 0.001) as in Fig. 1.

**Table 3:** Analysis of differences between medical and non-medical students’ levels of hypochondria and nosophobia using Student’s *t* test for independent data

	Medical students		Non-medical students		*T*	*P* value
	*M*	SD	*M*	SD		
Nosophobia	14.14	4.62	0.11	1.1	29.6	0.001
Hypochondria	7.87	4.4	1.43	2.6	13.62	0.001

*SD* Standard deviation, *N* Number of observations, *t* Value of Student's test, *M* Average

Examining the relation between the level of hypochondria and nosophobia symptoms in relation to the study year was another aspect of the investigation into the variations in the severity of these symptoms. The entire study group and smaller subgroups of students from the non-medical and medical faculties were examined in the analysis. Table 4 presents the findings. The scale of measurement of the variables led to the choice of the rank correlation coefficient.

**Table 4:** Summary of Spearman’s rho coefficient correlation between the study year and the severity of nosophobia and hypochondria symptoms across the whole group and by field of study.

Students	Variable under test	Year of study	
		*R*s	*p*
Non-medical	Nosophobia	− 0.05	0.312
	Hypochondria	− 0.23	0.082
Medical	Nosophobia	0. 47	0.031*
	Hypochondria	0.031	0.606
Total	Nosophobia	0.044	0.462
	Hypochondria	− 0.028	0.641

*p < 0.05

Examining the differences in the severity of nosophobia and hypochondria symptoms between males and females in both groups is a notable aspect of this research and examining whether it is related to the gender or to the year of study. A Mann–Whitney *U* test was applied for this reason. This analysis of the entire study group and the subgroups is presented in Table 5. Results that show that women achieve the same results in the level of hypochondria and nosophobia in the entire group of students of non-medical and medical faculties and individual groups of students can be presented for the groups that are not equal in size. For example, for nosophobia and hypochondria, women in non-medical fields had nonsignificant results (*p* > 0.05). When attempting to assess nosophobia in the medical students, no significant result for women was found (*p* > 0.05). Additionally, the results for male and female medical students presenting hypochondria symptoms were similar (*p* > 0.05).

**Table 5:** Summary of the Mann–Whitney *U* test correlation of the severity of symptoms of nosophobia and hypochondria between the studied females and males in the overall group and the division into research and control groups

Students	Variable under test	Sex						***U***	***P***
		Male			Female				
		*N*	*M*_rang_	*M*	*N*	*M*_rang_	*M*		
Non-medical	Nosophobia	43	49.63	13	43	48.5	13	1134.0	0.245
	Hypochondria	54	51.94	6	54	46.66	7		
Medical	Nosophobia	84	141.61	12	201	129.03	14	8325.5	0.063
	Hypochondria	84	129.03	6	201	148.8	7		
Total	Nosophobia	127	175.07	7	255	199.68	7	14106.5	0.017
	Hypochondria	127	172.59	7	255	200.92	7		

*N* Number of observations, *Me* Median, M_rang_—average rank, *U* Mann–Whitney U test value, *p*—significance

**Fig. 2:**
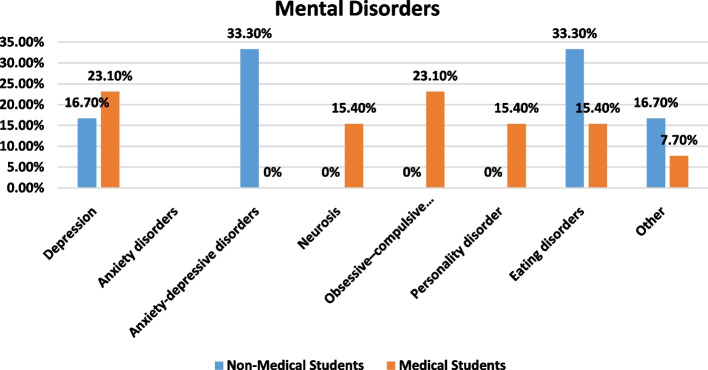
Percentage of survey respondents who indicated they had an admitted mental illness for both non-medical (blue) and medical (orange) faculties. (Blue) Number of non-medical students = 97. (Orange) Number of medical students = 285

The respondents were questioned on their mental health, whether they had seen a psychiatrist, and whether they were ready to start receiving psychiatric treatment. Results revealed that one third of the non-medical students (33.3) suffered from anxiety-depressive disorders. In a group of medical students, 23.1% declared having depression, and 23.1% declared having obsessive-compulsive disorder (23.1%) (Table 6). Mental disorders' percentages are represented in Fig. 2.

**Table 6:** Quantitative analysis of questions on admitted mental disorders

Mental disorders	Non-medical students	Medical students
Depression	1 (16.7%)	3 (23.1%)
Anxiety disorders		
Anxiety-depressive disorders	2 (33.3%)	0 (0%)
Neurosis	0 (0%)	2 (15.4%)
Obsessive-compulsive disorder	0 (0%)	3 (23.1%)
Personality disorder	0(0%)	2 (15.4%)
Eating disorders	2 (33.3%)	2 (15.4%)
Other	1 (16.7%)	1 (7.7%)

## Discussion

This study is one of the first to investigate the frequency of experiencing the symptoms of a specific disease after studying it and the fear of having such an illness in the future. We focused on the variations in behavior between non-medical and medical students in our sample of Menoufia University attendants. On the nosophobic scale, non-medical and medical students received different results. These results confirmed the common belief that medical students have exaggerated worries about their health. This myth started in the 1960s [9, 10]. This belief may be due to the nature of their study field because they study disease.

In contrast with our expectations, questions on anxiety side effects were observed more often among medical students than non-medical students. This finding is possibly caused by the lack of information among individuals who have not studied sufficient medical resources. This aspect leads to irrational fears of safe substances' or behaviors such as attempting to escape certain illnesses. Notably the increasing number of websites that provide non-evidence-based information plays a significant role in this phenomenon. In addition using reliable medical databases is challenging for non-medical students [3].

In our study, the fear of becoming ill correlates with the year of study. The higher the educational level of the students, the greater the level of nosophobia. However, the existing correlation in our data was relatively weak because in their early years, instead of clinical disorders, the medical study concentrates on pre-clinical sciences. . Later, their understanding of clinical conditions deepens. There was no correlation between the year studied and the level of nosophobia in non-medical students, corroborating the prior reason for the contrasting findings in medical students.

Surprisingly, the year of study showed no association with the level of hypochondria symptoms among non-medical and medical students. As a result, we could reasonably conclude that, despite an increase in nosophobia among medical students, the belief that they were sick did not rise. This finding is most likely due to these students' increased knowledge of the effective treatments for many diseases. Furthermore, because of the advanced knowledge gained through higher education levels, medical students can better understand their symptoms than students not studying medicine.

Our research also focused on additional aspects of mental health, such as the prevalence of mental health disorders. For example, our sample showed substantially higher rates of depression (16.7% for non-medical students, 23.1% for medical students) than those in a recent similar study in Poland (5.1–6%) [11]. In addition, anxiety disorder frequencies for the medical and non-medical students in this study (11% for non-medical and 14.14% for medical) were twice as much as those in the Polish study.

The next logical step was to determine if there was a correlation between concern over mental health and the number of students receiving psychiatric care. In our study, students treated for other mental problems reported higher anxiety levels and fear of being sick than those that did not—matching the results [12].

Another factor that can affect the fear of being sick is gender. For example, a study on 606 students from the Silesian region found that women are more likely than men to have a morbid fear for their health [13]. Additionally, we talk to a specialist doctor more often than men when they have concerns about their health.

A possible explanation for the differences between our study's findings and those in the literature is that college students have varying access to general health education because of widespread internet availability. Moreover, differences in personality traits might be found among generations of students [14]. Being a physician’s child is a factor that can also affect the study's results because medical students are often children of physicians [15]. In further research, a deeper examination than that conducted in this study of the differences in the severity of hypochondria and nosophobia symptoms between females and males in both groups, as well as a search for factors other than gender that may have influenced our findings, would yield additional insights. Stronger matching selection procedures than were used in this study should also be pursued, with criteria such as gender balance, socioeconomic situation, and access to health care being considered. Finally, our study’s findings provide insights into health policy for medical and non-medical students. As aforementioned, medical students have a high prevalence of mental health issues. Therefore, medical and other students should have access to mental health services that are accessible, cost-effective, and readily available for all students who require them.

## Conclusions

The outcomes of our study question the widely held idea that, compared with their non-medical peers, medical students are unduly concerned about their alleged well-being. For example, in Menoufia, the rate of individuals prone to anxiety and nosophobia side effects was higher among non-medical students than medical students. Women receiving therapy for various psychiatric diseases had more anxiety about their claimed well-being than their male counterparts. Finally, the prevalence of gloom and anxiety is substantial As a result; the sophisticated research in this area should increase.

## Data Availability

On reasonable request, the corresponding author, Huda A Sherif, will provide the data supporting this study's findings.
